# Longitudinal associations between violence exposure and adolescent conduct problems in a high‐adversity, South African setting

**DOI:** 10.1111/jcpp.70132

**Published:** 2026-02-11

**Authors:** Susan Swingler, Qing Han, Alexis E. Cullen, Stefani du Toit, Sarah Skeen, Jane Barlow, Mark Tomlinson

**Affiliations:** ^1^ Department of Social Policy and Intervention University of Oxford Oxford UK; ^2^ Institute of Population Research Peking University Beijing China; ^3^ Department of Clinical Neuroscience Karolinska Institutet Stockholm Sweden; ^4^ Department of Psychosis Studies, Institute of Psychiatry, Psychology & Neuroscience King's College London London UK; ^5^ Institute for Life Course Health Research, Department of Global Health Stellenbosch University Cape Town South Africa; ^6^ School of Nursing and Midwifery Queens University Belfast UK

**Keywords:** Childhood trauma, externalising problems, youth, prospective study

## Abstract

**Background:**

Violence exposure is a well‐established risk factor for adolescent conduct problems, yet longitudinal research in high‐adversity, low‐ and middle‐income countries (LMICs) remains limited. This study investigated whether early adolescent violence exposure predicts concurrent and longer‐term conduct problems, and explored potential bidirectional associations and sex differences in a peri‐urban South African community with high rates of poverty and violence.

**Methods:**

Data were drawn from the Thula Sana birth cohort (*n* = 357; 51.5% female), a longitudinal intervention study in Khayelitsha, South Africa. Adolescents were assessed at early (ages 12–14) and late adolescence (ages 16–19). Violence exposure was measured using adolescent self‐report. Conduct problems were measured using adolescent and caregiver report in early adolescence and adolescent self‐report in late adolescence. Multiple linear regressions tested cross‐sectional associations, and cross‐lagged panel models examined longitudinal and bidirectional associations, adjusting for contextual adversity and intervention status. Missing data were addressed using multiple imputation, and findings were confirmed through sensitivity analyses.

**Results:**

Violence exposure was associated with higher concurrent conduct problems in early adolescence (β = .15–.19, *p* < .01) and predicted higher conduct problems in late adolescence (β = .12–.14, *p* < .05). The reverse pathway, from conduct problems to subsequent violence exposure, was not significant (β = .08–.11, *p* > .05). Interaction analyses did not provide evidence that associations differed by sex.

**Conclusions:**

Violence exposure in early adolescence represents a prospective risk factor for conduct problems in a high‐adversity South African setting. Findings highlight the importance of early, contextually grounded violence prevention and the need for further research to test sex‐specific pathways and inform the development of gender‐responsive intervention strategies.

## Introduction

Childhood violence exposure is a major public health challenge in South Africa, where prevalence rates surpass global averages and inaction has been estimated at nearly 5% of the nation's GDP (Hsiao et al., 2018; Seedat, Van Niekerk, Jewkes, Suffla, & Ratele, 2009; Ward, Artz, Leoschut, Kassanjee, & Burton, 2018). Community‐based surveys show that South African children experience multiple forms of violence: 68.9% report psychological, physical and/or sexual abuse, over three quarters have witnessed family violence, and more than 90% have witnessed community violence, with 40.1% reporting direct threats or assaults (Kaminer, du Plessis, Hardy, & Benjamin, 2013; Meinck, Cluver, Boyes, & Loening‐Voysey, 2016; Richter, Mathews, Kagura, & Nonterah, 2018). Poly‐victimisation is common, with up to 93% of adolescents exposed to at least two forms of violence, and over one‐quarter experiencing multiple types simultaneously (Collings, Penning, & Valjee, 2014; Kaminer et al., 2013; Leoschut & Kafaar, 2017; Meinck et al., 2016). Sex differences have been documented, with boys more likely to experience physical and community violence and to report higher levels of poly‐victimisation compared with girls (Donenberg et al., 2020; Kaminer et al., 2013). Violence in South Africa persists due to structural and proximal risks that undermine protective support. These risks include poverty, unemployment, inadequate infrastructure, and norms condoning violence or gender hierarchy, as well as weak parent–child relationships, poor supervision, risky peers, unsafe environments and schools that condone corporal punishment or lack violence prevention strategies (Matzopoulos, Bowman, Mathews, & Myers, 2010; Ward et al., 2012).

Violence exposure has consistently been associated with greater concurrent and longer‐term conduct problems – including aggression and rule‐breaking – across diverse contexts (Evans, Davies, & DiLillo, 2008; Fowler, Tompsett, Braciszewski, Jacques‐Tiura, & Baltes, 2009; Vu, Jouriles, McDonald, & Rosenfield, 2016). Adolescents in South Africa are overrepresented among both victims and perpetrators of violence (Matzopoulos et al., 2010; Seedat et al., 2009; Ward et al., 2012). In a comparative study, De Vries, Davids, Mathews, and Aarø (2018) found that South African adolescents aged 12–16 had significantly higher conduct problem scores than adolescents from the UK, Australia and China. Moreover, a South African birth cohort study found that adolescent conduct problems predicted adverse adult outcomes, including criminality, substance use and intimate partner violence (Richter, Ahun, Besharati, Naicker, & Orri, 2021), thus perpetuating cycles of violence.

South African studies have found that violence exposure – whether direct or witnessed – is associated with adolescent conduct problems, with some evidence for stronger associations among boys (Donenberg et al., 2020; du Plessis, Kaminer, Hardy, & Benjamin, 2015). Critically, the cross‐sectional design of these studies limits inferences about directionality. Longitudinal research is needed to clarify whether violence exposure predicts the onset or escalation of adolescent conduct problems, whether conduct problems increase vulnerability to violence or whether these processes operate reciprocally over time. This is particularly important in high‐adversity contexts, where violence exposure is often chronic and structurally embedded, and where cumulative exposures may complicate assumptions about developmental directionality. In these settings, it is unclear whether associations between violence exposure and conduct problems are amplified by cumulative stress, attenuated due to the normalisation of violence or asymmetric in directionality due to structural constraints on adolescent agency. Adolescence is also a key intervention window, as conduct problems often emerge or escalate and behaviours remain malleable. Most existing longitudinal studies, however, come from high‐income countries (Evans et al., 2008; Fowler et al., 2009; Vu et al., 2016) and focus on unidirectional associations, with fewer exploring whether adolescent conduct problems may also increase the risk for future violence exposure (Mrug & Windle, 2009).

Various theoretical frameworks support the plausibility of pathways in both directions. General strain theory posits that stressors such as violence exposure may elicit anger and frustration that are externalised as conduct problems (Agnew, 1992), while social learning theory suggests that youth model the violent behaviours seen in family and community settings (Bandura, 1977). Gender socialisation may lead boys to externalise distress through aggression and girls to internalise it through anxiety or depression (Bussey & Bandura, 1999; Chaplin & Aldao, 2013; Zahn‐Waxler, Shirtcliff, & Marceau, 2008). Transactional models (Sameroff, 2009) further propose that violence exposure and conduct problems may reinforce one another over time, as early conduct problems increase the risk of harsh parenting, deviant peer affiliation and greater unsupervised time in unsafe environments (Farrell, Thompson, Curran, & Sullivan, 2020; Mrug & Windle, 2009). In high‐adversity settings such as South Africa, these transactional processes may be further shaped by structural constraints on adolescent agency and exposure. Accordingly, these frameworks provide a useful lens for understanding associations between violence exposure and conduct problems in adolescence, while motivating empirical tests of how such processes unfold in contexts of pervasive violence.

To investigate bidirectionality in longitudinal associations, some studies have used cross‐lagged panel model (CLPM) designs. Results are mixed: Some support bidirectional associations between violence exposure and conduct problems (Bauer et al., 2022; Boyes, Bowes, Cluver, Ward, & Badcock, 2014; Farrell et al., 2020; Mrug & Windle, 2009; Nobakht, Steinsbekk, & Wichstrøm, 2024), while others suggest that conduct problems are more likely to precede violence exposure (Esposito, Bacchini, Eisenberg, & Affuso, 2017; Mrug & Windle, 2009). Most of these studies have either not examined sex differences or report no significant differences. Few have been conducted in high‐adversity LMIC contexts, and those that have often focus on specific types of violence and include younger children in their samples (e.g. Bauer et al., 2022; Boyes et al., 2014). As a result, evidence remains limited on whether bidirectional associations between cumulative violence exposure and conduct problems across adolescence operate similarly in high‐adversity, LMIC contexts, where violence is pervasive and adolescent choice may be constrained.

The current study addresses these gaps by examining whether violence exposure in early adolescence is associated with concurrent and longer‐term conduct problems. We also explore possible reciprocal associations and sex differences in a high‐adversity, South African context. We hypothesised a positive cross‐sectional association between violence exposure and conduct problems in early adolescence, and that, after accounting for baseline conduct problems, violence exposure would predict elevated conduct problems in late adolescence. Given theoretical and empirical uncertainty regarding the magnitude and bidirectionality of associations in high‐adversity contexts, and our exploratory aims for the reverse pathway and sex differences, we did not specify hypotheses for these associations.

## Methods

### Participants and procedures

This study conducted a secondary analysis of data from the Thula Sana birth cohort, a longitudinal intervention study spanning nearly two decades (Cooper et al., 2009; Tomlinson, 2018). Participants were recruited from two Black, isiXhosa‐speaking communities in Khayelitsha, a peri‐urban settlement near Cape Town, South Africa, with high levels of poverty, crime and inadequate infrastructure (Freeman & McDonald, 2015; Levine, Swartz, & Rother, 2021; Seekings, 2013; Super, 2015).

The original cohort included 449 mother‐infant pairs, recruited during the mothers' third trimester of pregnancy as part of a randomised controlled trial (RCT) evaluating a perinatal home‐visiting intervention (Phase 1: 1999–2003, T1‐T5; see Figure S1). The trial assessed effects on maternal depression, mother–infant interactions and infant attachment security (Cooper et al., 2009). In Phase 2 (2012–2014, early adolescence, T6), 333 pairs (74.1% of the original trial) were followed up as part of the Saving Brains study, which examined long‐term effects on maternal mental health and adolescent cognitive development (Tomlinson et al., 2022). In Phase 3 (2019, late adolescence, T7–T9), 314 adolescents (70% of the original cohort) were re‐engaged in the Zifune RCT, aimed at reducing adolescent aggression and intimate partner violence through a cognitive‐behavioural intervention (Tomlinson, 2018). Data were collected at baseline (T7) and postintervention (T8, T9).

For the current study, adolescent participants were included if they provided data at early adolescence (ages 12–14; T6) and/or late adolescence (ages 16–19; T7), as these waves included measures of violence exposure and conduct problems. Analyses included both perinatal intervention and control group participants from the original trial. Covariates were drawn from T1, T2 and T6. T8 and T9 were excluded due to their focus on Zifune intervention outcomes. This allowed for a study sample of 357 adolescents and their caregivers.

### Measures

#### Violence exposure

At early adolescence (ages 12–14), violence exposure was assessed using an adapted 27‐item version of the Childhood Exposure to Community Violence (CECV) scale (Amaya‐Jackson, 1998; Cluver, Fincham, & Seedat, 2009), which has shown reliability in South African samples (α = .89; Boyes, Cluver, & Gardner, 2012). Adolescents self‐reported exposure to physical and sexual violence (e.g. robbery, assault, domestic violence, sexual abuse) on a three‐point scale (0 = Never, 1 = A few times, 2 = Many times). Higher summed scores indicate greater cumulative exposure. Only aggregate scores from the CECV were available. Previous analyses using the raw scores reported that adolescents were exposed to an average of 4.9 types of violence (Zimmerman et al., 2020), suggesting poly‐victimisation. At late adolescence (ages 16–19), a modified version assessed physical (but not sexual) violence; these scores are used as a dependent variable in bidirectional analyses.

#### Conduct problems

At early adolescence (ages 12–14), conduct problems were assessed using the self‐reported Strengths and Difficulties Questionnaire (SDQ; Goodman, 1997) and the caregiver‐reported Child Behaviour Checklist (CBCL; Achenbach & Rescorla, 2001). The SDQ conduct problem subscale includes five items on aggression and risk‐taking, scored on a three‐point scale (0 = Never, 1 = Somewhat true, 2 = Certainly true) and aggregated as a sum score. The CBCL externalising subscale includes 34 items on aggression and delinquency, also scored on a three‐point scale (0 = Not true, 1 = Somewhat true, 2 = Very true or often true) and summed as raw scores. Reliability in South African samples is high for the CBCL (α = .88–.94; Zieff, Fourie, Hoogenhout, & Donald, 2022) and moderate for the SDQ (α = .32–.71; Hoosen, Davids, De Vries, & Shung‐King, 2018), and both measures were used as multi‐informant assessment is recommended in early adolescence to capture a broader range of behaviours across different settings and reporters (De Los Reyes et al., 2015).

At late adolescence (ages 16–19), conduct problems were measured using the externalising subscale of the Youth Self Report (YSR; Achenbach & Rescorla, 2001), a 34‐item self‐report measure of aggression and rule‐breaking, rated on a three‐point scale (0 = Not true, 1 = Somewhat true, 2 = Very true or often true) and summed for a total score. The YSR has demonstrated reliability in South African settings (α = .71–.85; Zieff et al., 2022), and self‐report is generally considered appropriate for older adolescents, who can provide more accurate insight into their own behaviour (De Los Reyes et al., 2015).

#### Child sex

Child sex was reported by caregivers at T2.

#### Covariates

All analyses controlled for contextual adversity and original trial intervention group status. The index of contextual adversity (Fearon et al., 2017) was based on eight caregiver‐reported indicators at early adolescence (ages 12–14): overcrowding (household size above the highest quintile), lack of running water, lack of a toilet, lack of electricity, family members going a whole day without food due to lack of resources, primary caregiver unemployment, primary caregiver education limited to primary level and caregiver‐reported relationship breakdown with a partner or spouse. Indicators were dichotomised and averaged to create a continuous index (range: 0–1). Intervention status was coded as 0 (control group) or 1 (perinatal intervention group) at T1. Age was not included as a covariate because within‐wave age variability was limited (*SD*s < 1 year), and models already index developmental timing via assessment wave.

### Data analysis

The analysis plan was preregistered on the Open Science Framework (https://osf.io/ch2gq/) and deviations are detailed in Table S1. Analyses were conducted in Stata 18.5. Violence exposure and conduct problems scores for both time points were square root transformed prior to analyses.

#### Descriptive analyses

Descriptive statistics and bivariate correlations were calculated using complete cases. Independent *t*‐tests assessed differences in the continuous study variables based on sex and intervention status.

#### Missing data

Missing data were addressed using diagnostic analyses and multiple imputation to minimise bias and maximise power. Missing data percentages for key variables are shown in Table 1. Violence exposure and conduct problems at late adolescence (ages 16–19) had higher rates of missingness (up to 21.6%) compared with variables measured at early adolescence (ages 12–14) (<8%). To evaluate the missing at random (MAR) assumption, we ran multiple logistic regression analyses to predict missingness in violence exposure and conduct problems at late adolescence, using study variables at early adolescence (see Table S2). Higher contextual adversity in early adolescence predicted missing data for both outcomes, while missingness in conduct problems was more likely in the intervention group and less likely for those with higher caregiver‐reported conduct problems in early adolescence. These significant predictors were included as auxiliary variables in the imputation model and as covariates in subsequent analyses, supporting the MAR assumption.

**Table 1:** Descriptive statistics for continuous variables

Variable	*N*	Missing (%)	Mean	*SD*	Min	Max
Early adolescence (ages 12–14)						
Violence exposure (CECV)	333	6.72	6.39	5.49	0	29
Conduct problems (SDQ)	333	6.72	2.06	1.92	0	8
Conduct problems (CBCL)	330	7.56	13.23	12.84	0	76
Contextual adversity	332	7.00	0.30	0.20	0	1
Late adolescence (ages 16–19)						
Violence exposure (CECV)	280	21.57	4.87	5.28	0	33
Conduct problems (YSR)	314	12.04	9.33	7.38	0	38

Calculated using raw, untransformed scores.

We used multiple imputation (MI) by chained equations with predictive mean matching (PMM), chosen for its robustness to non‐normal distributions (Van Buuren, 2018). All study variables and predictors of missingness were included. Twenty imputed data sets were generated using ten nearest neighbours, consistent with recommendations that the number of imputations be similar to the proportion of incomplete cases when missing information is moderate (Van Buuren, 2018; White, Royston, & Wood, 2011). All analyses were run on pooled multiply imputed data sets, and continuous variables were standardised within each imputed dataset prior to analysis with coefficients reported as standardised effect sizes. Sensitivity analyses included complete‐case and full‐information maximum likelihood (FIML) approaches.

#### Multiple regression analyses

Multiple linear regression examined associations between violence exposure in early adolescence (ages 12–14) and concurrent conduct problems. Separate analyses were conducted for self‐ and caregiver‐reported conduct problems, with covariates entered simultaneously. Interaction terms tested moderation by sex. In the absence of statistically significant interactions, sex‐stratified analyses were conducted for descriptive purposes only across analytic approaches, including the CLPMs.

#### Cross‐lagged panel modelling

Longitudinal and bidirectional associations were examined using CLPM with maximum likelihood estimation. Separate models were run for self‐ and caregiver‐reported conduct problems in early adolescence (ages 12–14), rather than combining informants, to preserve informant‐specific variance and maintain parsimony in the longitudinal models. Each model included violence exposure and conduct problems in early adolescence as exogenous variables, and violence exposure and conduct problems in late adolescence as endogenous variables. Autoregressive and cross‐lagged paths were estimated, adjusting for contextual adversity. The path from violence exposure to later conduct problems was adjusted for perinatal intervention status to improve precision of estimated associations, given plausible effects of the intervention on behavioural outcomes. The reverse path was not adjusted, as intervention status was not expected to directly influence subsequent violence exposure. Covariances between violence exposure and conduct problems at each time point were freely estimated to account for shared risk factors and unmeasured influences.

## Results

### Descriptive analysis

Descriptive statistics and bivariate correlations are shown in Tables 1 and 2. Of the sample, 51.5% were female, and 48.2% had received the Thula Sana perinatal intervention. *T*‐tests indicated no significant differences by sex or intervention group for continuous study variables (see Tables S3 and S4). At the time of assessment, mean age was 12.76 years (*SD* = 0.66) at early adolescence (T6) and 17.41 years (*SD* = 0.54) at late adolescence (T7).

**Table 2:** Bivariable correlations for continuous variables

Variables	1	2	3	4	5	6
Early adolescence (ages 12–14)						
(1) Violence exposure (CECV)	1.0					
(2) Conduct problems (SDQ)	.20***	1.00				
(3) Conduct problems (CBCL)	.15***	.25***	1.00			
(4) Contextual adversity	−.06	.06	.13**	1.00		
Late adolescence (ages 16–19)						
(5) Violence exposure (CECV)	.32***	.15**	.19***	.08	1.00	
(6) Conduct problems (YSR)	.17***	.26***	.18***	.03	.34***	1.00

Calculated following square root transformation of violence exposure and conduct problem scores.

***p < .01, **p < .05, *p < .1.

### Cross‐sectional associations

Among the full sample, violence exposure in early adolescence was positively associated with concurrent adolescent problems, for both self‐reported (β = .19, *SE* = .05, *p* < .001) and caregiver‐reported (β = .15, *SE* = .06, *p* = .006) outcomes (Table 3). Standardised effects were similar in complete‐case analyses (β = .16–.21). Among covariates, only contextual adversity predicted higher caregiver‐reported conduct problems.

**Table 3:** Associations between violence exposure and concurrent self‐reported and caregiver‐reported problems in early adolescence (ages 12–14)

Independent variables	Model 1	Model 2
	β (*SE*)	β (*SE*)
Violence exposure	.19 (.05)***	.15 (.06)**
Contextual adversity	.08 (.06)	.14 (.06)*
Intervention group	−.15 (.11)	.09 (.11)

Conduct problems (T6) are self‐report (SDQ) in Model 1 and caregiver‐report (CBCL) in Model 2; β = standardised coefficients; *SE* = standard errors; continuous predictors are standardised; binary intervention group variable is entered unstandardised.

*p < .050, **p < .010, ***p < .001.

Interaction analyses did not indicate moderation by sex. Subsequent sex‐stratified analyses were conducted for descriptive purposes only (see Table S5). In these analyses, standardised coefficients for the association between violence exposure and concurrent conduct problems were numerically larger among girls (β = .21–.27) and lower among boys (β = .09–.12) across informants. By contrast, coefficients for contextual adversity were numerically larger among boys (β = .17–.21) and lower among girls (β = −.01 to .08).

These results indicate a robust association between early adolescent violence exposure and concurrent conduct problems, with no support for associations differing by sex.

### Longitudinal and bidirectional associations

In the full sample, violence exposure in early adolescence was positively associated with conduct problems in late adolescence (Model 1: β = .12, *SE* = .06, *p* = .037; Model 2: β = .14, *SE* = .06, *p* = .013; Table 4). The reverse pathway – from conduct problems to later violence exposure – was not statistically significant (Model 1: β = .08, *SE* = .06, *p* = .169; Model 2: β = .11, *SE* = 0.06, *p* = .070; Table 4). These pathways are illustrated in Figure S2. Sensitivity analyses using complete cases and FIML analyses confirmed these effects were small and consistent across informants (see Tables S6 and S7).

**Table 4:** Cross‐lagged panel model examining the longitudinal, bidirectional associations between violence exposure and conduct problems

Structural paths	Model 1	Model 2
	β (*SE*)	β (*SE*)
Autoregressive paths		
Violence exposure (T6 → T7)	.30 (.06)***	.29 (.06)***
Conduct problems (T6 → T7)	.22 (.06)***	.14 (.06)*
Cross‐lagged paths		
Violence exposure (T6) → Conduct problems (T7)	.12 (.06)*	.14 (.06)*
Conduct problems (T6) → Violence exposure (T7)	.08 (.06)	.11 (.06)
Covariate paths		
Contextual adversity (T6) → Violence exposure (T7)	.06 (.06)	.05 (.06)
Contextual adversity (T6) → Conduct problems (T7)	.00 (.06)	.00 (.06)
Intervention group (T1) → Conduct problems (T7)	.12 (.10)	.08 (.11)
Covariances		
Violence exposure (T6) ↔ Conduct problems (T6)	.19 (.06)**	.14 (.06)*
Violence exposure (T7) ↔ Conduct problems (T7)	.26 (.06)***	.26 (.06)***

Conduct problems (T6) are self‐report (SDQ) in Model 1 and caregiver‐report (CBCL) in Model 2; β = standardised coefficient; SE = standard error; for continuous predictors, β represents the change in the outcome associated with a one standard deviation increase in the predictor. For the binary treatment group variable, β represents the standardised mean difference in the outcome between intervention and control groups; T6 = early adolescence (ages 12–14), T7 = late adolescence (ages 16–19).

*p < .050, **p < .010, ***p < .001.

To explore whether these longitudinal associations differed by sex, we first tested an interaction between violence exposure and sex in multiple regression models predicting later conduct problems, adjusting for baseline conduct problems. The interaction was not significant. Subsequent sex‐stratified analyses were conducted for descriptive purposes only (see Table S8) and showed similar magnitudes of the violence‐to‐conduct pathway across sexes, with standardised coefficients ranging from β = .13–.15 among boys and β = .10–.14 among girls across informants. Sex‐stratified models also examined the reverse pathway from conduct problems to subsequent violence exposure. In these analyses, conduct‐to‐violence coefficients were small in both sexes, with estimates near zero among boys (β = .02–.09) and numerically larger among girls (β = .14 across informants).

Taken together, results indicate that among the full sample, violence exposure in early adolescence robustly predicts conduct problems in late adolescence.

## Discussion

This is one of the few longitudinal studies in high‐adversity LMICs to have examined reciprocal links between violence exposure and conduct problems across adolescence. Consistent with our hypothesis, violence exposure in early adolescence (ages 12–14) predicted higher concurrent and later conduct problems (at ages 16–19; after adjusting for baseline conduct problems). We further explored bidirectional associations and sex differences, given evidence that violence exposure and conduct problems may be mutually reinforcing and may differ by sex due to social norms, behavioural expression and exposure contexts. The following sections discuss the study findings in relation to existing literature and consider implications for intervention and future research.

In early adolescence, greater violence exposure was cross‐sectionally associated with higher conduct problems in both adolescent self‐reports and caregiver reports. Compared to meta‐analyses conducted primarily in high‐income countries reporting effects in the moderate range (Evans et al., 2008; Fowler et al., 2009; *d* = 0.45–0.50 and *r* = 0.20–0.40, respectively), these associations were somewhat smaller in relative magnitude, though our findings align with research in South African communities (e.g. β = .12–.26; Donenberg et al., 2020; du Plessis et al., 2015). One plausible explanation is that in high‐adversity settings where violence is pervasive, violence may be regarded as a normative aspect of life, attenuating the psychological and behavioural consequences (Dawes, Kropiwnicki, Kafaar, & Richter, 2005; Mrug & Windle, 2010). Alternatively, elevated baseline levels of conduct problems may reduce the relative impact of violence exposure by restricting variance. However, variability in conduct problem scores in our sample suggests this is not solely responsible for the modest effect sizes observed.

Interaction tests did not provide evidence that cross‐sectional associations between violence exposure and conduct problems differed by sex, and sex‐stratified analyses were presented to support hypothesis generation. In these analyses, the associations between violence exposure and conduct problems in early adolescence appeared smaller among boys, with larger point estimates for cumulative contextual adversities (e.g. food insecurity, overcrowding, caregiver unemployment) as a predictor of conduct problems. This pattern aligns with general strain theory (Agnew, 1992), which posits that persistent strains such as chronic material hardship can lead to negative affect and behavioural externalisation. These descriptive findings raise the hypothesis that in high‐adversity contexts, boys' conduct problems may be more closely related to cumulative, ongoing socioeconomic stressors than to recent exposure to violence. Among girls, point estimates for the violence‐conduct problem association appeared larger in magnitude after adjusting for covariates, including cumulative contextual adversity. While it could be hypothesised that girls exhibit conduct problem behaviour in response to violence exposure, it is also plausible that girls who display such behaviours – potentially perceived as violating strict gender norms in patriarchal contexts – may be more vulnerable to punitive responses including gender‐based violence and marginalisation from protective networks (Jewkes & Abrahams, 2002; Jewkes, Penn‐Kekana, & Rose‐Junius, 2005; Kim & Motsei, 2002; Richter et al., 2021).

We used cross‐lagged panel modelling (CLPM) to examine the temporal and bidirectional associations. In the full sample, early adolescent violence exposure was associated with greater conduct problems in late adolescence. This extends previous research and is consistent with prior studies using CLPM with both child and adolescent samples in high‐income (Esposito et al., 2017; Farrell et al., 2020; Mrug & Windle, 2009) and LMIC contexts (Bauer et al., 2022; Boyes et al., 2014). We show that in a high‐adversity LMIC context, adolescence remains a sensitive period during which cumulative violence exposure can trigger or exacerbate conduct problems. Given the high rates of conduct problems among South African adolescents (du Plessis et al., 2015), even modest increases can have serious long‐term social and economic consequences (Richter et al., 2021). By contrast, the reverse longitudinal pathway – from early adolescent conduct problems to later violence exposure – was not statistically significant in the full sample. This contrasts with some previous research and may reflect contextual factors in Khayelitsha, where structural factors sustaining violence may diminish the influence of individual behaviour on subsequent exposure (Seedat et al., 2009). It is also possible that the study lacked power to detect small reciprocal associations, as reported in larger LMIC cohort studies (Bauer et al., 2022; Boyes et al., 2014).

Interaction tests did not provide evidence that longitudinal associations between violence exposure and conduct problems differed by sex. Accordingly, sex‐stratified CLPMs were interpreted cautiously and are presented to support hypothesis generation rather than inference. Specifically, the pattern of point estimates highlights potential sex‐specific hypotheses that could be tested in future, adequately powered studies. Among boys, estimates for the violence‐to‐conduct pathway were small and together with the small cross‐sectional associations suggest the possibility that they may show a delayed behavioural response to violence exposure. Among girls, estimates for the conduct‐to‐violence pathway appeared larger in magnitude, which – together with robust cross‐sectional associations – generates the hypothesis that behavioural difficulties and violence exposure may become mutually reinforcing for adolescent girls in this context. In patriarchal settings, conduct problems in girls may be viewed as transgressive and could increase their vulnerability to punitive or violent responses, including gender‐based violence and social marginalisation (Jewkes & Abrahams, 2002; Jewkes et al., 2005; Kim & Motsei, 2002; Richter et al., 2021). Future research is needed in high‐adversity, LMIC contexts to test these sex‐specific hypotheses using larger samples and with greater attention to social norms and context.

Several limitations warrant consideration. First, although the sample size was sufficient for detecting the modest effect of violence exposure on subsequent conduct problems, it may have been underpowered to detect smaller effects, particularly for the reverse pathway, interaction analyses and in sex‐stratified models. Larger samples are needed to robustly test these pathways. Relatedly, formal corrections for multiple testing were not applied, which may increase the risk of Type I error; however, analyses were theory‐driven, limited in number and prespecified, and exploratory sex‐stratified findings were interpreted cautiously. Second, the cumulative measure of violence exposure, while valuable for capturing overall burden, did not differentiate between type, severity, context or proximity of exposures. Disaggregating specific forms of violence and poly‐victimisation profiles may reveal more nuanced developmental pathways and potential sex differences. Third, the two‐wave design limits causal interpretation and the ability to track trajectories of violence exposure and conduct problems across adolescence. At least three measurement points are required to implement analytical approaches capable of disentangling within‐ and between‐person processes (e.g. random‐intercept CLPM; Usami, Murayama, & Hamaker, 2019). Fourth, potential protective factors (e.g. social support, resilience, community resources) or additional risks (e.g. deviant peer affiliations, inequitable gender norms) were not examined but likely influence associations, especially in high‐adversity contexts. Finally, the generalisability of these findings may be limited to similar high‐adversity, peri‐urban South African settings, and further research in other LMIC contexts is warranted.

Despite its limitations, this study has notable strengths and adds to the evidence that violence exposure in early adolescence is a modifiable and prospective risk factor for conduct problems in high‐adversity LMIC settings. A major strength of this study is its implementation within a non‐Western, high‐adversity setting where sustained longitudinal data collection across adolescence is logistically challenging. Retention over nearly two decades in a context characterised by socioeconomic deprivation, high mobility and ongoing exposure to violence reflects considerable commitment from participating families and the sustained efforts of local research teams. Our findings further reinforce the importance of adolescence as a window for violence prevention, when behavioural consequences may escalate, but when intervention opportunities remain. These findings support calls for multisectoral strategies that target risk and protective factors across individual, family, school and community levels (Seedat et al., 2009; Ward et al., 2012). Group‐based interventions – including parenting support, cognitive‐therapy based approaches and school‐based interventions promoting social–emotional learning and violence prevention – show promise in resource‐constrained settings (Ward et al., 2012). While exploratory, these findings highlight the importance of future research explicitly testing whether pathways linking violence exposure and conduct problems differ by sex, and how such differences – if supported – might inform the development of gender‐responsive intervention strategies. Addressing broader structural drivers, such as socioeconomic inequality and youth unemployment, is also essential to reducing the burden of violence exposure in South African communities (Matzopoulos et al., 2010; Seedat et al., 2009). Together, these approaches may help reduce the overall ‘dose’ of violence exposure and its lasting consequences for youth development.

## Conclusion

Drawing on longitudinal data from a South African birth cohort, this study found that violence exposure in early adolescence robustly predicted both concurrent and later conduct problems in a high‐adversity setting. Although we found no evidence for a reverse pathway from conduct problems to later violence exposure in the full sample, exploratory analyses highlight hypotheses regarding potential sex‐specific pathways that warrant testing in future research. These findings reinforce adolescence as a key intervention window and emphasise the need for multisectoral strategies, with future work needed to clarify whether and how gender‐responsive approaches may be most effective in high‐adversity settings.

## Ethical considerations

Prior to participation, adult caregivers provided written informed consent for both their own and their child's participation, and adolescents provided written assent. Ethical approvals were obtained for all phases: University of Reading Ethics and Research Committee (approval date: 21/10/1999; ref: 99/20) and University of Cape Town Medical School Research Ethics Committee for Phase 1 (approval date: 02/02/1998; ref: 180/97), and Stellenbosch University Health Research Ethics Committee for Phase 2 (approval date: 2012; ref: S12/04/113) and Phase 3 (approval date: 29/11/2017; ref: N17/10/094).

**Key points**

**What's known?**

- Violence exposure is a well‐established risk factor for conduct problems, yet few longitudinal studies have examined this association during adolescence in low‐ and middle‐income countries.

**What's new?**

- This study demonstrates that violence exposure in early adolescence predicts both concurrent and later conduct problems over a four‐year period in a South African birth cohort.
- Findings highlight adolescence as a critical window for prevention, with early violence exposure contributing to lasting behavioural risk.
- No evidence was found for a reverse pathway from conduct problems to later violence exposure. Exploratory analyses did not identify sex differences but suggest hypotheses for future testing.

**What's relevant?**

- Findings support contextually grounded, multisectoral violence prevention in adolescence, with future research needed to inform gender‐responsive approaches in high‐adversity settings.

## Supporting information


**Figure S1.** Phases of Thula Sana Birth Cohort Study Data Collection.
**Figure S2.** Path diagram of cross‐lagged panel models with standardised (β) coefficients from multiple imputation analyses.
**Table S1.** Deviations from the Preregistered Analysis Plan and Justifications.
**Table S2.** Multiple Logistic Regression Analyses Examining Missingness in Variables in Late Adolescence (ages 16–19).
**Table S3.** Descriptive statistics and *t*‐test comparisons of continuous study variables by sex.
**Table S4.** Descriptive statistics and *t*‐test comparisons of continuous study variables by perinatal intervention group status.
**Table S5.** Associations between violence exposure and concurrent self‐reported and caregiver‐reported problems in early adolescence (ages 12–14), stratified by sex.
**Table S6.** Comparison of CLPM Model 1 across multiple imputation, FIML and complete‐case analyses.
**Table S7.** Comparison of CLPM Model 2 across multiple imputation, FIML and complete‐case analyses.
**Table S8.** Cross‐lagged panel model examining the longitudinal, bidirectional associations between violence exposure and conduct problems, stratified by sex.

## Data Availability

Data used in this study are not publicly available due to ethical restrictions but can be requested from Mark Tomlinson at the Institute for Life Course Health Research, Department of Global Health, Stellenbosch University.

## References

[jcpp70132-bib-0001] Achenbach, T.M. , & Rescorla, L.A. (2001). Manual for the ASEBA school‐age forms & profiles. Burlington, VT: University of Vermont, Research Center for Children, Youth & Families.

[jcpp70132-bib-0002] Agnew, R. (1992). Foundation for a general strain theory of crime and delinquency. Criminology, 30, 47–88.

[jcpp70132-bib-0003] Amaya‐Jackson, L. (1998). Child's exposure to violence checklist. Adopted from Richter's things I've seen and heard. Trauma evaluation, treatment and research program. Durham: Center for Child and Family Health.

[jcpp70132-bib-0004] Bandura, A. (1977). Social learning theory. Englewood Cliffs, NJ: Prentice‐Hall.

[jcpp70132-bib-0005] Bauer, A. , Fairchild, G. , Halligan, S.L. , Hammerton, G. , Murray, J. , Santos, I.S. , … & Matijasevich, A. (2022). Harsh parenting and child conduct and emotional problems: Parent‐ and child‐effects in the 2004 Pelotas Birth Cohort. European Child & Adolescent Psychiatry, 31, 1–11. 10.1007/s00787-021-01759-w PMC9343272 33738622

[jcpp70132-bib-0006] Boyes, M.E. , Bowes, L. , Cluver, L.D. , Ward, C.L. , & Badcock, N.A. (2014). Bullying victimisation, internalising symptoms, and conduct problems in south African children and adolescents: A longitudinal investigation. Journal of Abnormal Child Psychology, 42, 1313–1324. 24882504 10.1007/s10802-014-9888-3

[jcpp70132-bib-0007] Boyes, M.E. , Cluver, L.D. , & Gardner, F. (2012). Psychometric properties of the Child PTSD Checklist in a community sample of South African children and adolescents. PLoS One, 7, e46905. 23056523 10.1371/journal.pone.0046905 PMC3463511

[jcpp70132-bib-0008] Bussey, K. , & Bandura, A. (1999). Social cognitive theory of gender development and differentiation. Psychological Review, 106, 676–713. 10560326 10.1037/0033-295x.106.4.676

[jcpp70132-bib-0009] Chaplin, T.M. , & Aldao, A. (2013). Gender differences in emotion expression in children: A meta‐analytic review. Psychological Bulletin, 139, 735–765. 23231534 10.1037/a0030737 PMC3597769

[jcpp70132-bib-0010] Cluver, L. , Fincham, D.S. , & Seedat, S. (2009). Post‐traumatic stress in AIDS‐orphaned children exposed to high levels of trauma: The protective role of perceived social support. Journal of Traumatic Stress, 22, 106–112. 19319917 10.1002/jts.20396

[jcpp70132-bib-0011] Collings, S.J. , Penning, S.L. , & Valjee, S.R. (2014). Lifetime poly‐victimization and posttraumatic stress disorder among school‐going adolescents in Durban, South Africa. Journal of Psychiatry, 17, 5.

[jcpp70132-bib-0012] Cooper, P.J. , Tomlinson, M. , Swartz, L. , Landman, M. , Molteno, C. , Stein, A. , … & Murray, L. (2009). Improving quality of mother‐infant relationship and infant attachment in socioeconomically deprived community in South Africa: Randomised controlled trial. BMJ, 338, b974. 19366752 10.1136/bmj.b974 PMC2669116

[jcpp70132-bib-0013] Dawes, A. , Kropiwnicki, Z.D.S. , Kafaar, Z. , & Richter, L. (2005). Corporal punishment of children: A South African national survey. Cape Town: Human Sciences Research Council.

[jcpp70132-bib-0014] De Los Reyes, A. , Augenstein, T.M. , Wang, M. , Thomas, S.A. , Drabick, D.A.G. , Burgers, D.E. , & Rabinowitz, J. (2015). The validity of the multi‐informant approach to assessing child and adolescent mental health. Psychological Bulletin, 141, 858–900. 25915035 10.1037/a0038498 PMC4486608

[jcpp70132-bib-0015] De Vries, P.J. , Davids, E.L. , Mathews, C. , & Aarø, L.E. (2018). Measuring adolescent mental health around the globe: Psychometric properties of the self‐report Strengths and Difficulties Questionnaire in South Africa, and comparison with UK, Australian and Chinese data. Epidemiology and Psychiatric Sciences, 27, 369–380. 28112065 10.1017/S2045796016001207 PMC6998978

[jcpp70132-bib-0016] Donenberg, G. , Naidoo, P. , Kendall, A. , Emerson, E. , Ward, C.L. , Kagee, A. , … & Mackesy‐Amiti, M.E. (2020). Pathways from witnessing community violence to mental health problems among South African adolescents. South African Medical Journal, 110, 145–153. 32657687 10.7196/SAMJ.2020.v110i2.13929 PMC9327528

[jcpp70132-bib-0017] du Plessis, B. , Kaminer, D. , Hardy, A. , & Benjamin, A. (2015). The contribution of different forms of violence exposure to internalizing and externalizing symptoms among young south African adolescents. Child Abuse & Neglect, 45, 80–89. 25804436 10.1016/j.chiabu.2015.02.021

[jcpp70132-bib-0018] Esposito, C. , Bacchini, D. , Eisenberg, N. , & Affuso, G. (2017). Effortful control, exposure to community violence, and aggressive behavior: Exploring cross‐lagged relations in adolescence. Aggressive Behavior, 43, 588–600. 28603851 10.1002/ab.21717

[jcpp70132-bib-0019] Evans, S.E. , Davies, C. , & DiLillo, D. (2008). Exposure to domestic violence: A meta‐analysis of child and adolescent outcomes. Aggression and Violent Behavior, 13, 131–140.

[jcpp70132-bib-0020] Farrell, A.D. , Thompson, E.L. , Curran, P.J. , & Sullivan, T.N. (2020). Bidirectional relations between witnessing violence, victimization, life events, and physical aggression among adolescents in urban schools. Journal of Youth and Adolescence, 49, 1309–1327. 32008134 10.1007/s10964-020-01204-2 PMC7242153

[jcpp70132-bib-0021] Fearon, R.M.P. , Tomlinson, M. , Kumsta, R. , Skeen, S. , Murray, L. , Cooper, P.J. , & Morgan, B. (2017). Poverty, early care, and stress reactivity in adolescence: Findings from a prospective, longitudinal study in South Africa. Development and Psychopathology, 29, 449–464. 28401838 10.1017/S0954579417000104 PMC5659183

[jcpp70132-bib-0022] Fowler, P.J. , Tompsett, C.J. , Braciszewski, J.M. , Jacques‐Tiura, A.J. , & Baltes, B.B. (2009). Community violence: A meta‐analysis on the effect of exposure and mental health outcomes of children and adolescents. Development and Psychopathology, 21, 227–259. 19144232 10.1017/S0954579409000145

[jcpp70132-bib-0023] Freeman, L. , & McDonald, C. (2015). Mapping Khayelitsha—The complexities of everyday policing in a high crime area. South African Quarterly, 53, 27–37.

[jcpp70132-bib-0024] Goodman, R. (1997). The strengths and difficulties questionnaire: A research note. Journal of Child Psychology and Psychiatry, 38, 581–586. 9255702 10.1111/j.1469-7610.1997.tb01545.x

[jcpp70132-bib-0025] Hoosen, N. , Davids, E.L. , De Vries, P.J. , & Shung‐King, M. (2018). The Strengths and Difficulties Questionnaire (SDQ) in Africa: A scoping review of its application and validation. Child and Adolescent Psychiatry and Mental Health, 12, 6. 29344084 10.1186/s13034-017-0212-1 PMC5765647

[jcpp70132-bib-0026] Hsiao, C. , Fry, D. , Ward, C.L. , Ganz, G. , Casey, T. , Zheng, X. , & Fang, X. (2018). Violence against children in South Africa: The cost of inaction to society and the economy. BMJ Global Health, 3, e000573. 10.1136/bmjgh-2017-000573 PMC5838395 29515918

[jcpp70132-bib-0027] Jewkes, R. , & Abrahams, N. (2002). The epidemiology of rape and sexual coercion in South Africa: An overview. Social Science & Medicine, 55, 1231–1244. 12365533 10.1016/s0277-9536(01)00242-8

[jcpp70132-bib-0028] Jewkes, R. , Penn‐Kekana, L. , & Rose‐Junius, H. (2005). ‘If they rape me, I can't blame them’: Reflections on gender in the social context of child rape in South Africa and Namibia. Social Science & Medicine, 61, 1809–1820. 15913860 10.1016/j.socscimed.2005.03.022

[jcpp70132-bib-0029] Kaminer, D. , du Plessis, B. , Hardy, A. , & Benjamin, A. (2013). Exposure to violence across multiple sites among young south African adolescents. Peace and Conflict: Journal of Peace Psychology, 19, 112–124.

[jcpp70132-bib-0030] Kim, J. , & Motsei, M. (2002). “Women enjoy punishment”: Attitudes and experiences of gender‐based violence among PHC nurses in rural South Africa. Social Science & Medicine, 54, 1243–1254. 11989960 10.1016/s0277-9536(01)00093-4

[jcpp70132-bib-0031] Leoschut, L. , & Kafaar, Z. (2017). The frequency and predictors of poly‐victimisation of South African children and the role of schools in its prevention. Psychology, Health & Medicine, 22(Sup1), 81–93. 10.1080/13548506.2016.1273533 28073295

[jcpp70132-bib-0032] Levine, S. , Swartz, A. , & Rother, H.A. (2021). Invisible harms: Humans as waste, toxic layering, and childhood poisoning in Cape Town, South Africa. In Bodies of knowledge: Children and childhoods in health and affliction . Stellenbosch, South Africa: African Sun Media.

[jcpp70132-bib-0033] Matzopoulos, R. , Bowman, B. , Mathews, S. , & Myers, J. (2010). Applying upstream interventions for interpersonal violence prevention: An uphill struggle in low‐ to middle‐income contexts. Health Policy, 97, 62–70. 20381189 10.1016/j.healthpol.2010.03.003

[jcpp70132-bib-0034] Meinck, F. , Cluver, L.D. , Boyes, M.E. , & Loening‐Voysey, H. (2016). Physical, emotional and sexual adolescent abuse victimisation in South Africa: Prevalence, incidence, perpetrators and locations. Journal of Epidemiology and Community Health, 70, 910–916. 26962202 10.1136/jech-2015-205860 PMC5013157

[jcpp70132-bib-0035] Mrug, S. , & Windle, M. (2009). Bidirectional influences of violence exposure and adjustment in early adolescence: Externalizing behaviors and school connectedness. Journal of Abnormal Child Psychology, 37, 611–623. 19199024 10.1007/s10802-009-9304-6

[jcpp70132-bib-0036] Mrug, S. , & Windle, M. (2010). Prospective effects of violence exposure across multiple contexts on early adolescents' internalizing and externalizing problems: Violence exposure across contexts. Journal of Child Psychology and Psychiatry, 51, 953–961. 20331489 10.1111/j.1469-7610.2010.02222.x PMC3857691

[jcpp70132-bib-0037] Nobakht, H.N. , Steinsbekk, S. , & Wichstrøm, L. (2024). Reciprocal relations between interparental aggression and symptoms of oppositional defiant and conduct disorders: A seven‐wave cohort study of within‐family effects from preschool to adolescence. Journal of Child Psychology and Psychiatry, 65, 753–763. 37786360 10.1111/jcpp.13903

[jcpp70132-bib-0038] Richter, L.M. , Ahun, M.N. , Besharati, S. , Naicker, S.N. , & Orri, M. (2021). Adolescent mental health problems and adult human capital: Findings from the South African Birth to Twenty Plus Cohort at 28 years of age. Journal of Adolescent Health, 69, 782–789. 10.1016/j.jadohealth.2021.04.017 PMC8552796 34059430

[jcpp70132-bib-0039] Richter, L.M. , Mathews, S. , Kagura, J. , & Nonterah, E. (2018). A longitudinal perspective on violence in the lives of South African children from the Birth to Twenty Plus cohort study in Johannesburg‐Soweto. South African Medical Journal, 108, 181–186. 30004360 10.7196/SAMJ.2018.v108i3.12661

[jcpp70132-bib-0040] Sameroff, A. (Ed.). (2009). The transactional model of development: How children and contexts shape each other. Washington, DC: American Psychological Association. 10.1037/11877-000

[jcpp70132-bib-0041] Seedat, M. , Van Niekerk, A. , Jewkes, R. , Suffla, S. , & Ratele, K. (2009). Violence and injuries in South Africa: Prioritising an agenda for prevention. The Lancet, 374, 1011–1022. 10.1016/S0140-6736(09)60948-X 19709732

[jcpp70132-bib-0042] Seekings, J. (2013). Economy, society and municipal services in Khayelitsha. Report for the Commission of Inquiry into allegations of police inefficiency in Khayelitsha and a breakdown in relations between the community and the police in Khayelitsha. Centre for Social Science Research, University of Cape Town.

[jcpp70132-bib-0043] Super, G. (2015). Violence and democracy in Khayelitsha, governing crime through the ‘community’. International Journal of Security and Development, 4, 1–20. 10.5334/sta.ft

[jcpp70132-bib-0044] Tomlinson, M. (2018). Does an adolescent programme delivered to teenagers who took part in a mother‐infant intervention reduce interpersonal violence? [data set]. 10.1186/ISRCTN11940364

[jcpp70132-bib-0045] Tomlinson, M. , Skeen, S. , Melendez‐Torres, G.J. , Hunt, X. , Desmond, C. , Morgan, B. , … & Fearon, P. (2022). First 1,000 days: Enough for mothers but not for children? Long‐term outcomes of an early intervention on maternal depressed mood and child cognitive development: Follow‐up of a randomised controlled trial. Journal of Child Psychology and Psychiatry, 63, 261–272. 34227113 10.1111/jcpp.13482

[jcpp70132-bib-0046] Usami, S. , Murayama, K. , & Hamaker, E.L. (2019). A unified framework of longitudinal models to examine reciprocal relations. Psychological Methods, 24, 637–657. 30998041 10.1037/met0000210

[jcpp70132-bib-0047] Van Buuren, S. (2018). Flexible imputation of missing data (2nd edn). New York: Chapman and Hall/CRC. 10.1201/9780429492259

[jcpp70132-bib-0048] Vu, N.L. , Jouriles, E.N. , McDonald, R. , & Rosenfield, D. (2016). Children's exposure to intimate partner violence: A meta‐analysis of longitudinal associations with child adjustment problems. Clinical Psychology Review, 46, 25–33. 27136293 10.1016/j.cpr.2016.04.003

[jcpp70132-bib-0049] Ward, C.L. , van der Merwe, A. , & Dawes, A. . (2012). Youth violence: Sources and solutions in South Africa. Cape Town, South Africa: UCT Press.

[jcpp70132-bib-0050] Ward, C.L. , Artz, L. , Leoschut, L. , Kassanjee, R. , & Burton, P. (2018). Sexual violence against children in South Africa: A nationally representative cross‐sectional study of prevalence and correlates. The Lancet Global Health, 6, e460–e468. 29530424 10.1016/S2214-109X(18)30060-3

[jcpp70132-bib-0051] White, I.R. , Royston, P. , & Wood, A.M. (2011). Multiple imputation using chained equations: Issues and guidance for practice. Statistics in Medicine, 30, 377–399. 21225900 10.1002/sim.4067

[jcpp70132-bib-0052] Zahn‐Waxler, C. , Shirtcliff, E.A. , & Marceau, K. (2008). Disorders of childhood and adolescence: Gender and psychopathology. Annual Review of Clinical Psychology, 4, 275–303. 10.1146/annurev.clinpsy.3.022806.091358 18370618

[jcpp70132-bib-0053] Zieff, M.R. , Fourie, C. , Hoogenhout, M. , & Donald, K.A. (2022). Psychometric properties of the ASEBA Child Behaviour Checklist and Youth Self‐Report in sub‐Saharan Africa—A systematic review. Acta Neuropsychiatrica, 34, 167–190. 35466902 10.1017/neu.2022.5

[jcpp70132-bib-0054] Zimmerman, A. , Halligan, S. , Skeen, S. , Morgan, B. , Fraser, A. , Fearon, P. , & Tomlinson, M. (2020). PTSD symptoms and cortisol stress reactivity in adolescence: Findings from a high adversity cohort in South Africa. Psychoneuroendocrinology, 121, 104846. 32919210 10.1016/j.psyneuen.2020.104846

