# Supplementary material for: Longitudinal associations between violence exposure and adolescent conduct problems in a high‐adversity, South African setting
Source: J Child Psychol Psychiatry. 2026 Feb 11;67(8):1244–53. doi: 10.1111/jcpp.70132 (PMC13341350; doi:10.1111/jcpp.70132)
Supplement: Supplementary file 1 — Figure S1. Phases of Thula Sana Birth Cohort Study Data Collection. Figure S2. Path diagram of cross‐lagged panel models with standardised (β) coefficients from multiple imputation analyses. Table S1. Deviations from the Preregistered Analysis Plan and Justifications. Table S2. Multiple Logistic Regression Analyses Examining Missingness in Variables in Late Adolescence (ages 16–19). Table S3. Descriptive statistics and t‐test comparisons of continuous study variables by sex. Table S4. Descriptive statistics and t‐test comparisons of continuous study variables by perinatal intervention group status. Table S5. Associations between violence exposure and concurrent self‐reported and caregiver‐reported problems in early adolescence (ages 12–14), stratified by sex. Table S6. Comparison of CLPM Model 1 across multiple imputation, FIML and complete‐case analyses. Table S7. Comparison of CLPM Model 2 across multiple imputation, FIML and complete‐case analyses. Table S8. Cross‐lagged panel model examining the longitudinal, bidirectional associations between violence exposure and conduct problems, stratified by sex. [file JCPP-67-1244-s001.docx]

# Longitudinal Associations Between Violence Exposure and Adolescent Conduct Problems in a High-Adversity, South African Setting

# Supporting Information

*Figure S1.* Phases of Thula Sana birth cohort study data collection.


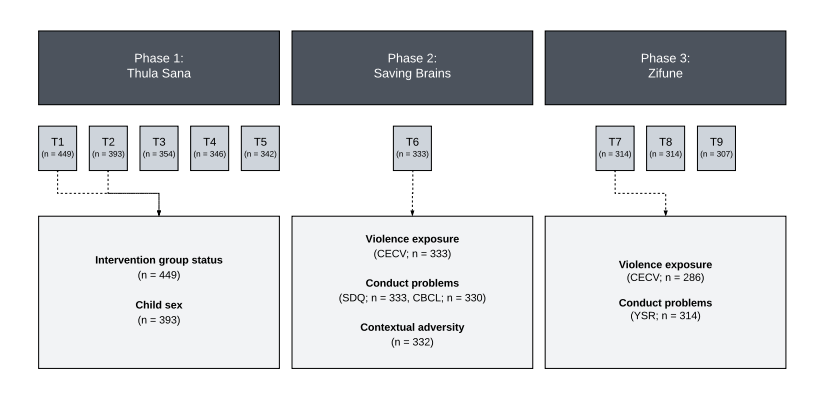


## Table S1

*Deviations from the Pre-Registered Analysis Plan and Justifications*

| **Planned element** | **Preregistered plan** | **Deviation** | **Justification** |
| --- | --- | --- | --- |
| Cortisol mediation analyses | Examine cortisol response (AUCg, AUCi) as a mediator between violence exposure and conduct problems. | Does not include cortisol analyses or mediation analyses at all. | Cortisol mediation hypothesis is being tested in a separate, stand-a-lone study. |
| Data analysis software and procedures | Analyses planned in R, using the *lavaan* package for cross-lagged panel models and the *mice* package for multiple imputation. | Analyses conducted in Stata 18.5. Multiple imputation implemented using Stata's PMM instead of the mice package in R. | Practical considerations concerning efficiency and ease of troubleshooting in Stata 18.5. |
| Handling of missing data | Planned Little’s test to assess missing data patterns (MCAR, MAR, MNAR). | Instead of Little’s test, logistic regression was used to explore predictors of missing data under the MAR assumption. | Little’s test provides only an omnibus test, whereas logistic regression identifies predictors of missingness, which can be incorporated into the imputation model and included as covariates in subsequent analyses. |
| Data transformation | No transformation of violence exposure and conduct problems variables. | Violence exposure and conduct problems variables were square-root transformed. Descriptive statistics, however, are reported using the raw, untransformed data. | Initial exploration of data distributions revealed moderate positive skewness in violence exposure and conduct problem variables, for which simple square-root transformations effectively reduced skewness and improved normality. |
| Covariate adjustment | Adjusting for child sex as a covariate. | Child sex was excluded as a covariate in models. | The study tested sex by violence exposure interactions and conducted subgroup analyses by sex. Adjusting for child sex in the main effect models would have been unnecessary and conceptually inconsistent. |
| Covariate adjustment | Adjusting for intervention status in both cross-lagged pathways (violence exposure to conduct problems, and conduct problems to violence exposure). | Adjusting for intervention status only in the violence exposure to conduct problems pathway, while the reverse path was not adjusted. | Adjustment was prioritized for the violence exposure to conduct problems path, as this was the primary pathway of interest, and because intervention status may more plausibly impact adolescent behavioural outcomes (via early caregiving effects) than adolescent violence exposure. |

## Table S2

*Multiple Logistic Regression Analyses Examining Missingness in Variables in Late Adolescence (ages 16-19)*

| Dependent variable | Independent variables | *OR* | *SE* | *z* | *p* | *95% CIs of OR* | |
| --- | --- | --- | --- | --- | --- | --- | --- |
|  |  |  |  |  |  | *Lower* | *Upper* |
| Missing violence exposure (CECV) | Violence exposure (T6) | 0.84 | 0.10 | -1.50 | 0.134 | 0.68 | 1.05 |
|  | Conduct problems (SDQ; T6) | 1.35 | 0.23 | 1.76 | 0.079 | 0.97 | 1.88 |
|  | Conduct problems (CBCL; T6) | 0.90 | 0.07 | -1.29 | 0.196 | 0.77 | 1.06 |
|  | Sex | 0.95 | 0.26 | -0.18 | 0.854 | 0.56 | 1.63 |
|  | Contextual adversity | 8.42 | 5.98 | 3.00 | 0.003 | 2.09 | 33.89 |
|  | Perinatal intervention group | 1.48 | 0.41 | 1.42 | 0.156 | 0.86 | 2.56 |
| Missing conduct problems (YSR) | Violence exposure (T6) | 0.79 | 0.11 | -1.68 | 0.092 | 0.60 | 1.04 |
|  | Conduct problems (SDQ) | 1.39 | 0.30 | 1.53 | 0.126 | 0.91 | 2.12 |
|  | Conduct problems (CBCL) | 0.79 | 0.08 | -2.32 | 0.020 | 0.64 | 0.96 |
|  | Sex | 0.56 | 0.20 | -1.65 | 0.099 | 0.28 | 1.12 |
|  | Contextual adversity | 25.51 | 23.05 | 3.59 | 0.000 | 4.34 | 149.84 |
|  | Perinatal intervention group | 2.36 | 0.87 | 2.33 | 0.020 | 1.14 | 4.87 |

Note: *OR* = Odds ratio; *SE* = standard error; *z* = *z*-statistic; *p* = *p*-value; *CI* = confidence interval; T6 = early adolescence (ages 12-14).

## Table S3

*Descriptive Statistics and T-Test Comparisons of Continuous Study Variables by Sex*

| Variable | Boys | | | Girls | | |  |  |
| --- | --- | --- | --- | --- | --- | --- | --- | --- |
|  | *N* | *M* | *SD* | *N* | *M* | *SD* | *t* | *p* |
| Early adolescence (ages 12-14) |  |  |  |  |  |  |  |  |
| Violence exposure (CECV) | 166 | 6.62 | 5.59 | 167 | 6.16 | 5.38 | 0.64 | 0.525 |
| Conduct problems (SDQ) | 166 | 2.02 | 1.94 | 167 | 2.10 | 1.90 | -0.56 | 0.576 |
| Conduct problems (CBCL) | 165 | 13.63 | 13.48 | 165 | 12.82 | 12.20 | 0.39 | 0.697 |
| Contextual adversity | 166 | 0.30 | 0.20 | 166 | 0.31 | 0.19 | -0.19 | 0.851 |
| Late adolescence (ages 16-19) |  |  |  |  |  |  |  |  |
| Violence exposure (CECV) | 135 | 5.08 | 5.15 | 145 | 4.68 | 5.41 | 0.91 | 0.365 |
| Conduct problems (YSR) | 148 | 9.35 | 7.22 | 166 | 9.31 | 7.54 | 0.34 | 0.737 |
| Note: Means (*M*) and standard deviations (*SD*) calculated using raw scores, and two-tailed independent-samples *t*-tests were conducted following square-root transformation of violence exposure and conduct problems scores. | | | | | | | | |

## Table S4

*Descriptive Statistics and T-Test Comparisons of Continuous Study Variables by Perinatal Intervention Group Status*

|  | Control group | | | Intervention group | | |  |  |
| --- | --- | --- | --- | --- | --- | --- | --- | --- |
| Variable | *N* | *M* | *SD* | *N* | *M* | *SD* | *t* | *p* |
| Early adolescence (ages 12-14) |  |  |  |  |  |  |  |  |
| Violence exposure (CECV) | 162 | 6.15 | 4.90 | 171 | 6.61 | 6.00 | -0.25 | 0.801 |
| Conduct problems (SDQ) | 162 | 2.14 | 1.84 | 171 | 1.99 | 1.99 | 1.11 | 0.268 |
| Conduct problems (CBCL) | 161 | 12.43 | 11.77 | 169 | 13.99 | 13.77 | -1.12 | 0.264 |
| Contextual adversity | 161 | 0.29 | 0.19 | 171 | 0.32 | 0.20 | -1.43 | 0.155 |
| Late adolescence (ages 16-19) |  |  |  |  |  |  |  |  |
| Violence exposure (CECV) | 140 | 5.05 | 5.13 | 140 | 4.69 | 5.44 | 0.91 | 0.362 |
| Conduct problems (YSR) | 157 | 9.03 | 6.52 | 157 | 9.62 | 8.15 | -0.42 | 0.677 |
| Note: Means (*M*) and standard deviations (*SD*) calculated using raw scores, and two-tailed independent-samples *t*-tests were conducted following square-root transformation of violence exposure and conduct problems scores. | | | | | | | | |

## Table S5

*Associations Between Violence Exposure and Concurrent Self-Reported and Caregiver-Reported Problems in Early Adolescence (Ages 12-14), Stratified by Sex*

|  | **Model 1** | | **Model 2** | |
| --- | --- | --- | --- | --- |
| **Independent variables** | **Boys (n = 173)** | **Girls (n = 184)** | **Boys (n = 173)** | **Girls (n = 184)** |
|  | β *(SE)* | β *(SE)* | β *(SE)* | β *(SE)* |
| Violence exposure | 0.12 (0.08) | 0.27 (0.08)** | 0.09 (0.08) | 0.21 (0.08)** |
| Contextual adversity | 0.17 (0.08)* | -0.01 (0.08) | 0.21 (0.08)** | 0.08 (0.09) |
| Intervention group | -0.16 (0.15) | -0.13 (0.15) | *0.19 (0.16)* | 0.01 (0.15) |

Note: Conduct problems (T6) are self-report (SDQ) in Model 1 and caregiver-report (CBCL) in Model 2; β = standardised coefficients; *SE* = standard errors; Continuous predictors are standardised; The binary intervention group variable is entered unstandardised; **p* < 0.050, ***p* < 0.010, ****p* < 0.001.

*Figure S2.* Path Diagram of Cross-Lagged Panel Models with Standardised (β) Coefficients from Multiple Imputation Analyses. Model 1 includes self-reported conduct problems and Model 2 includes caregiver-reported conduct problems at early adolescence (ages 12-14, T6). Solid lines indicate statistically paths (*p* < 0.05), and dashed lines indicate non-significant paths. Standard errors and exact *p*-values are reported in Tables S5-S6.


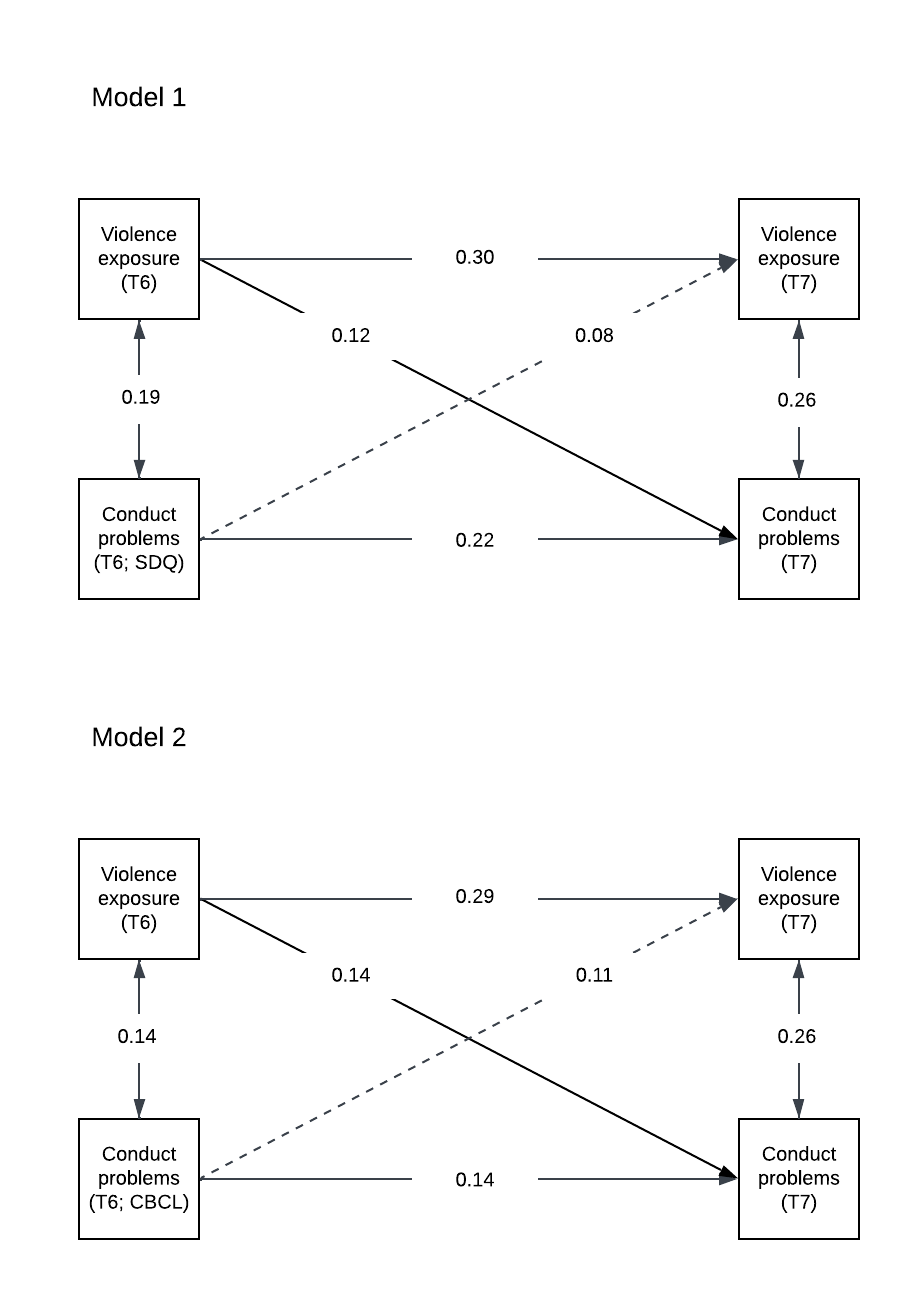


## Table S6

*Comparison of CLPM Model 1 Across Multiple Imputation, FIML, and Complete Case Analyses*

| **Structural paths** | **MI (n = 357)** | **FIML (n = 357)** | **CC (n = 259)** |
| --- | --- | --- | --- |
|  | β *(SE)* | β *(SE)* | β *(SE)* |
| **Autoregressive paths** |  |  |  |
| Violence exposure (T6 → T7) | 0.30 (0.06)*** | 0.31 (0.06)*** | 0.29 (0.06)*** |
| Conduct problems (T6 → T7) | 0.22 (0.06)*** | 0.24 (0.06)*** | 0.25 (0.06)*** |
| **Cross-lagged paths** |  |  |  |
| Violence exposure (T6) → Conduct problems (T7) | 0.12 (0.06)* | 0.12 (0.06)* | 0.12 (0.06) |
| Conduct problems (T6) → Violence exposure (T7) | 0.08 (0.06) | 0.09 (0.06) | 0.09 (0.06) |
| **Covariate paths** |  |  |  |
| Contextual adversity (T6) → Violence exposure (T7) | 0.06 (0.06) | 0.06 (0.05) | 0.07 (0.06) |
| Contextual adversity (T6) → Conduct problems (T7) | 0.00 (0.06) | 0.01 (0.06) | 0.02 (0.06) |
| Intervention group (T1) → Conduct problems (T7) | 0.12 (0.10) | 0.06 (0.05) | 0.06 (0.06) |
| **Covariances** |  |  |  |
| Violence exposure (T6) ↔ Conduct problems (T6) | 0.19 (0.06)** | 0.20 (0.05)*** | 0.21 (0.06)** |
| Violence exposure (T7) ↔ Conduct problems (T7) | 0.26 (0.06)*** | 0.29 (0.05)*** | 0.28 (0.06)*** |

Note: Model 1 conduct problems at T6 are self-report (SDQ); T6 = early adolescence (ages 12-14), T7 = late adolescence (ages 16-19); MI = multiple imputation; FIML = full information maximum likelihood estimation; CC = complete case analysis; β = standardised coefficient*; SE* = standard error; Continuous predictors are standardised in all models; Binary treatment group variable is not standardised in the MI analyses, but is standardised in the FIML and CC analyses; **p* < 0.05, ***p* < 0.01, ****p* < 0.001.

## Table S7

*Comparison of CLPM Model 2 Across Multiple Imputation, FIML, and Complete Case Analyses*

| **Structural paths** | **MI (n = 357)** | **FIML (n = 357)** | **CC (n = 259)** |
| --- | --- | --- | --- |
|  | β *(SE)* | β *(SE)* | β *(SE)* |
| **Autoregressive paths** |  |  |  |
| Violence exposure (T6 → T7) | 0.29 (0.06)*** | 0.31 (0.06)*** | 0.29 (0.06)*** |
| Conduct problems (T6 → T7) | 0.14 (0.06)* | 0.16 (0.06)** | 0.20 (0.06)*** |
| **Cross-lagged paths** |  |  |  |
| Violence exposure (T6) → Conduct problems (T7) | 0.14 (0.06)* | 0.15 (0.06)* | 0.14 (0.06)* |
| Conduct problems (T6) → Violence exposure (T7) | 0.11 (0.06) | 0.13 (0.06)* | 0.14 (0.09)* |
| **Covariate paths** |  |  |  |
| Contextual adversity (T6) → Violence exposure (T7) | 0.05 (0.06) | 0.05 (0.05) | 0.05 (0.06) |
| Contextual adversity (T6) → Conduct problems (T7) | 0.00 (0.06) | 0.01 (0.06) | 0.00 (0.06) |
| Intervention group (T1) → Conduct problems (T7) | 0.08 (0.11) | 0.03 (0.05) | 0.02 (0.06) |
| **Covariances** |  |  |  |
| Violence exposure (T6) ↔ Conduct problems (T6) | 0.14 (0.06)* | 0.15 (0.06)* | 0.16 (0.06)** |
| Violence exposure (T7) ↔ Conduct problems (T7) | 0.26 (0.06)*** | 0.29 (0.05)*** | 0.27 (0.06)*** |

Note: Model 2 conduct problems are caregiver-report (CBCL); T6 = early adolescence (ages 12-14), T7 = late adolescence (ages 16-19); MI = multiple imputation; FIML = full information maximum likelihood estimation; CC = complete case analysis; β = standardised coefficient; *SE* = standard error; Continuous predictors are standardised in all models; Binary treatment group variable is not standardised in the MI analyses, but is standardised in the FIML and CC analyses; **p* < 0.05, ***p* < 0.01, ****p* < 0.001.

## Table S8

*Cross-Lagged Panel Model Examining the Longitudinal, Bidirectional Associations Between Violence Exposure and Conduct Problems, Stratified by Sex*

| **Structural paths** | **Model 1** | | **Model 2** | |
| --- | --- | --- | --- | --- |
|  | **Boys (n = 173)** | **Girls (n = 184)** | **Boys (n = 173)** | **Girls (n = 184)** |
|  | β *(SE)* | β *(SE)* | β *(SE)* | β *(SE)* |
| **Autoregressive paths** |  |  |  |  |
| Violence exposure (T6 → T7) | 0.29 (0.08)*** | 0.29 (0.09)** | 0.28 (0.08)*** | 0.30 (0.08)** |
| Conduct problems (T6 → T7) | 0.21 (0.12)* | 0.25 (0.09)** | 0.11 (0.08) | 0.17 (0.08)* |
| **Cross-lagged paths** |  |  |  |  |
| Violence exposure (T6) → Conduct problems (T7) | 0.13 (0.08) | 0.10 (0.09) | 0.15 (0.08) | 0.14 (0.09) |
| Conduct problems (T6) → Violence exposure (T7) | 0.02 (0.08) | 0.14 (0.08) | 0.09 (0.09) | 0.14 (0.09) |
| **Covariate paths** |  |  |  |  |
| Contextual adversity (T6) → Violence exposure (T7) | 0.11 (0.08) | 0.03 (0.09) | 0.09 (0.09) | 0.02 (0.09) |
| Contextual adversity (T6) → Conduct problems (T7) | -0.01 (0.08) | 0.02 (0.08) | 0.00 (0.08) | 0.01 (0.08) |
| Intervention group (T1) → Conduct problems (T7) | 0.15 (0.14) | 0.08 (0.15) | 0.10 (0.15) | 0.05 (0.15) |
| **Covariances** |  |  |  |  |
| Violence exposure (T6) ↔ Conduct problems (T6) | 0.13 (0.08) | 0.26 (0.08)** | 0.09 (0.08) | 0.20 (0.08)** |
| Violence exposure (T7) ↔ Conduct problems (T7) | 0.28 (0.08)*** | 0.24 (0.08)** | 0.28 (0.08)*** | 0.25 (0.08)** |

Note: Conduct problems (T6) are self-report (SDQ) in Model 1 and caregiver-report (CBCL) in Model 2; T6 = early adolescence (ages 12-14), T7 = late adolescence (ages 16-19); β = standardised coefficient*; SE* = standard error; Continuous predictors are standardised; Binary treatment group variable is not standardised; **p* < 0.050, ***p* < 0.010, ****p* < 0.001.
